# A Potential Role and Contribution of Androgens in Placental Development and Pregnancy

**DOI:** 10.3390/life11070644

**Published:** 2021-07-01

**Authors:** Agata M. Parsons, Gerrit J. Bouma

**Affiliations:** Animal Reproduction and Biotechnology Laboratory, Department of Biomedical Sciences, Colorado State University, Fort Collins, CO 80523, USA; agata.parsons_aubone@colostate.edu

**Keywords:** androgens, androgen receptors, placenta, trophoblast cells, pregnancy

## Abstract

Successful pregnancy requires the establishment of a highly regulated maternal–fetal environment. This is achieved through the harmonious regulation of steroid hormones, which modulate both maternal and fetal physiology, and are critical for pregnancy maintenance. Defects in steroidogenesis and steroid signaling can lead to pregnancy disorders or even fetal loss. The placenta is a multifunctional, transitory organ which develops at the maternal–fetal interface, and supports fetal development through endocrine signaling, the transport of nutrients and gas exchange. The placenta has the ability to adapt to adverse environments, including hormonal variations, trying to support fetal development. However, if placental function is impaired, or its capacity to adapt is exceeded, fetal development will be compromised. The goal of this review is to explore the relevance of androgens and androgen signaling during pregnancy, specifically in placental development and function. Often considered a mere precursor to placental estrogen synthesis, the placenta in fact secretes androgens throughout pregnancy, and not only contains the androgen steroid nuclear receptor, but also non-genomic membrane receptors for androgens, suggesting a role of androgen signaling in placental function. Moreover, a number of pregnancy disorders, including pre-eclampsia, gestational diabetes, intrauterine growth restriction, and polycystic ovarian syndrome, are associated with abnormal androgen levels and androgen signaling. Understanding the role of androgens in the placenta will provide a greater understanding of the pathophysiology of pregnancy disorders associated with androgen elevation and its consequences.

## 1. Androgens and Pregnancy

Steroid hormones are important for the maintenance of normal pregnancy. For example, progesterone (P4), from the corpus luteum and mainly from the placenta, has important functions in the process of implantation and parturition by promoting endometrial decidualization and inhibiting smooth muscle contractility. In fact, P4 inhibts prostaglandin (PG) formation, which helps maintain myometrial quiescence and prevents the onset of uterine contractions [1]. Estrogens (E2), primarily from the placenta, are key to the promotion of embryo implantation and angiogenesis in the placenta [2]. In addition to estrogen and progesterone, androgen levels increase in maternal plasma during pregnancy, suggesting a role for this hormone in a successful outcome to pregnancy.

Androgens are steroid hormones that play important roles in female reproductive physiology, including pregnancy. In woman, ovaries, adrenal glands, adipose tissue and the placenta are all sources of androgens; i.e., dehydroepiandrosterone (DHEA), dehydroepiandrosterone sulfate (DHEAS), dihydrotestosterone (DHT), and testosterone (T) [3]. DHEA is the primary androgen secreted by adrenal glands, and is converted to androstenedione and T, which, in turn, can be converted by CYP19A1 (aromatase) to E2 [4]. In addition, T can be converted to the more potent DHT by 5α-reductase. T and DHT are the only two androgens that bind and activate the androgen receptor (AR) [5].

Although the placenta has the same function in all mammals, i.e., allowing for the exchange of nutrients and gasses and secrete steroid hormones, there are differences in androgen synthesis and secretion during pregnancy [6]. For example, in pregnant cows, the plasma concentration of T increases markedly during the last trimester of gestation. [7]. In horses, T levels are high early and late in pregnancy [8]. The first peak in T secretion is caused by the corpus luteum. This is caused by embryonic secretion of equine chorionic gonadotropin acting on the corpus luteum and stimulating steroidogenesis. In addition, fetal gonads in both male and females are initially very large and secrete T, before reducing in size, which coincides with a drop in maternal T plasma levels. The placenta in mares has little capacity to synthesize T itself, as it lacks 17α-hydroxylase (CYP17A1). Therefore, DHEA and T, mostly from fetal gonads, serve as precursors for E2 synthesis, forming a true feto–placental unit [8]. Finally, maternal serum levels of testosterone nearly tripled, reaching ~0.11 ng/mL near term, in pregnant sheep [9].

Studies in women also revealed an increase in maternal T levels during pregnancy. Androgens are secreted during the first trimester of pregnancy and are approximately three-fold higher near term than T levels seen in non-pregnant women [10]. Contrary to the mare, human placentas contain CYP17A1, and are thus able to synthesize androgens *de novo* [11]. Overall, these data indicate that the placenta is a source of testosterone during pregnancy.

Although the increase in androgen levels is expected in normal pregnancies, an excess in the production of androgens has been reported in pregnancy complications and disorders associated with placental dysfunction. For example, polycystic ovarian syndrome (PCOS) is accompanied by elevated levels of androgen, and can result in pregnancy complications such as pre-eclampsia. Preeclampsia is a human pregnancy syndrome characterized by hypertension and proteinuria after 20 weeks of gestation [12]. Although the exact cause of preeclampsia is still unclear, it is characterized by impaired placental development, during which there is a reduction in spiral artery re-modeling and generally shallow invasion, leading to fetal growth restriction. Recent studies have suggested androgens as an emerging contributor to the pathology of preeclampsia [13]. Women with preeclampsia have elevated androgen levels and AR gene expression, and it has been hypothesized that androgens affect vascular function, and vascular smooth muscle is involved in the development of preeclampsia. Moreover, it is postulated that an abnormal levels of androgens negatively impacts placental angiogenesis and/or alters trophoblast cell proliferation and differentiation [13,14], although the exact mechanisms are unclear.

## 2. AR Localization in the Mammalian Placentas

In addition to being a source of androgens during pregnancy, the placenta is also a target for androgens, in that all mammalian placental types contain AR (see below).

**Figure 1:**
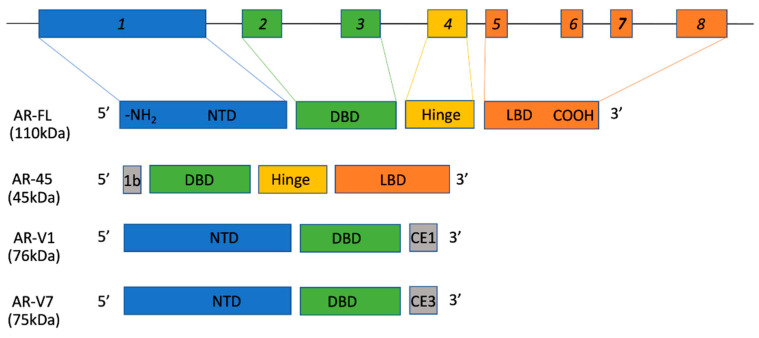
Schematic illustration of the AR gene and it’s major splice variants. The AR gene, shown on top, contains 8 exons and codes for a 919 amino acid protein. Like other members belonging to the steroid hormone receptor family, the full-length AR protein (AR-FL) contains several functional domains required for normal protein function. Exon 1 encodes the N-terminal domain (NTD), which also contains a transactivation domain. Exons 2 and 3 encode for the DNA-binding domain, which interact with a androgen response element (ARE) in promoter regions of target genes. Exon 4 contains the hinge domain, which encodes nuclear localization signals that facilitate cytoplasmic to nuclear translocation. Finally, exons 5–8 encode the ligand binding domain (LBD) and an additional trasnactivation domain. In addition to AR-FL, the placenta expresses three more different splice variants: AR-45 which does not express the NTD, AR-V1 and AR-V7 which do not contain the hinge region and the LBD. 1b = exon 1b. CE1/3 = cryptic exons1/3.

Humans have a discoid hemochorial placenta, which is formed of a single disc containing many chorionic villi lined with a fetal chorionic epithelium, which is bathed in maternal blood [15]. During placentation, some chorionic villi attach and invade the maternal endometrial epithelium and replace uterine arterial endothelial cells (spiral aterial remodeling), enabling increased blood flow to the chorionic villi. AR protein was found in the nuclei and the cytosol of the syncytiotrophoblast layer, but not in either the cytosol or nuclei of fetal membranes [16]. AR has been detected in first-trimester and term placenta, with positive AR nuclear and cytoplasmic staining in syncytiotrophoblasts and stromal cells [17]. More recently, AR splice variants have been identified in human and sheep placentas [18,19]. Specifically, four AR variants, full-length AR (AR-FL), AR-V1, AR-V7 and AR-45, were identified in both human and sheep placentas, although their cell-specific localization was not described [18,19]. (Figure 1). The presence of these AR variants was further investigated in placentas during impaired pregnancies associated with maternal asthma. Maternal asthma during pregnancy is associated with fetal growth restriction and preterm delivery, and impacts female fetuses (which are smaller) more than male fetuses. Interestingly, sex-specific expression of placental AR variants was reported, with male placenta (i.e., placenta associated with a male fetus) from asthmatic pregnancies showing a significant reduction in the nuclear expression of AR-FL, AR-V7 and AR-V1, but an increase in the nuclear expression of AR-45, while female placenta showed no changes [20]. Altered AR signaling in the presence of these isoforms may explain the differential response of developing fetuses to asthma in mothers during pregnancy between males and females.

**Figure 2:**
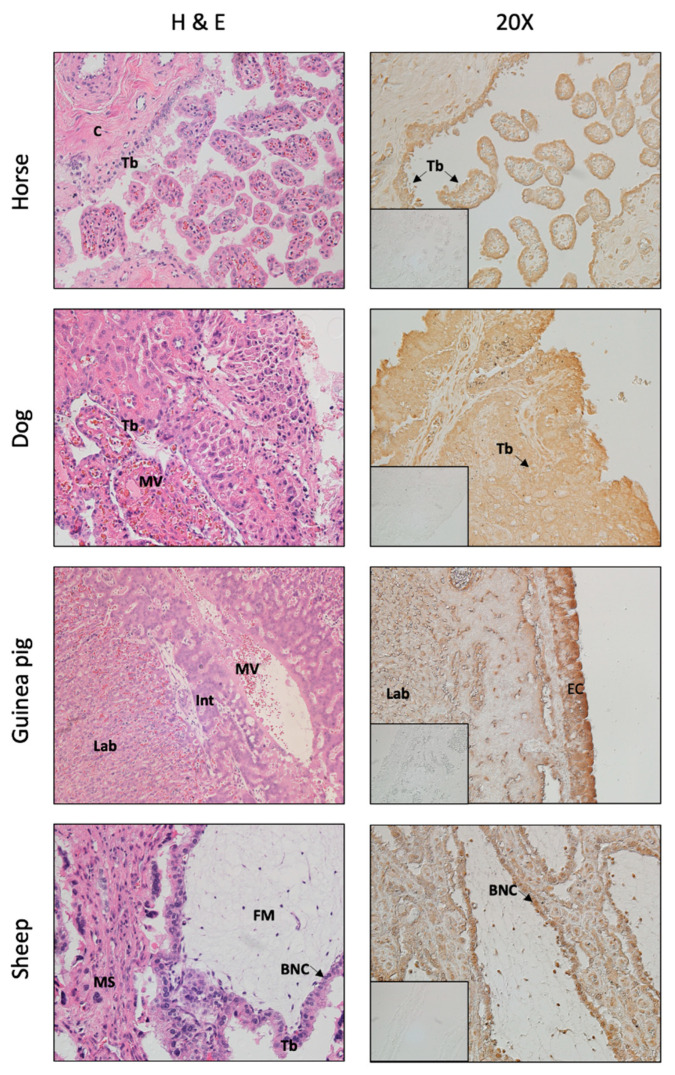
AR localization in 4 mammalian placenta types. Tb = trophoblast, MV = maternal vessel, MS = maternal stroma, FM = fetal mesenchyme, EC = epithelial cells. BNC = binucleate cell, C = chorion, Lab = labyrinth, Int = interlobar areas. Horse (diffuse) and dog (zonary) placenta show positive AR staining in the trophoblast cells. Guinea pig (discoid) placenta have faint staining in the labyrinth zone and more intense staining in the epithelial cells. Sheep (cotyledonary) placentome contains AR in trophoblast cells and in maternal and fetal stroma. Insert on right panel indicates negative control, and images are taken at 20X.

Ruminants have a cotyledonary, synepitheliochorial placenta, which consists of multiple distinct areas where maternal caruncle and fetal cotyledon together form a placentome where nutrient and gas exchange occurs [15]. In this type of placenta, fetal and maternal blood are separated by six layers (fetal endothelial cells, fetal connective tissue, fetal chorion epithelium, maternal endometrial epithelium, maternal connective tissue, and maternal endothelial cells). This is called synepitheliochorial because the endometrial epithelium is modified by the fusion of endometrial epithelial cells with fetal trophoblast cells, forming binucleate cells and eventually a syncytium. In addition, multinucleated trophoblast giant cells (TGC) are thought to remove endometrial epithelial cells and fuse and contribute to the syncytial trophoblast layer [21]. AR is present in sheep placentomes with nuclear staining predominantly in TGC and uninucleate trophoblast cells, as well as fetal and maternal stromal cells, and caruncular epithelial cells during late gestation [22] (Figure 2). Again, all four AR variants were identified in cytoplasmatic and nuclear fractions from sheep placentomes, including AR-FL, AR-v1, AR-v7 and AR-45. In bovine placentomes, AR staining has been observed in caruncular and cotyledonary stromal cells (exclusively nuclear), and caruncular epithelium and trophoblast cells. Prominent staining was found throughout gestation in invasive TGC [23].

Horses have a diffuse, epitheliochorial placenta, in which chorionic villi are in contact with the uterine endometrium throughout the entire surface of the allantochorion. Similar to ruminants and their synepithliochorial (cotyledonary) placenta, this consists of six layers separating maternal from fetal blood [24]. The main difference in the horse (i.e., diffuse placenta) is the lack of fusion between fetal trophoblast cells and maternal endometrial epithelial cells and formation of a syncytium. Studies in our lab revealed AR immunolocalization in horse placenta, with positive staining in the chorionic lining and trophoblast cells (Figure 2).

Dogs have a zonary endotheliochorial placenta, which surrounds the chorionic sack [25]. During placentation, the maternal endometrial epithelium is invaded and fetal chorionic epithelial cells come into contact with maternal endothelial cells. AR is distributed ubiquitously and found in cytotrophoblast and syncytiotrophoblast, in decidual cells and in vascular endothelial cells (*tunica intima*) (Figure 2).

Guinea pigs contain a discoidal, labyrinthine, hemo-monochorial placenta, where the placental disc contains spaces with maternal blood, and fetal capillaries lined by fetal endothelium, which is separated from the syncytiotrophoblast by a basal lamina [26]. AR presented a faint staining in the labyrinth zone and an intense staining in the epithelial cells of the placental disc (Figure 2).

## 3. Androgen Signaling: Genomic and Non-Genomic Signaling Pathways

AR resides on the X chromosomes and belongs to the steroid hormone receptor family of transcription factors. As described in Figure 1, the AR gene contains eight exons, which yield a 919 amino acid protein. Like other steroid receptors, the AR protein has N-terminal and C-terminal domains containing a transactivation function region that interacts with coregulatory proteins to enhance transcriptional activation. Furthermore, as a ligand-dependent transcription factor, it has a DNA binding domain that consists of two distinct zinc-fingers which interact with a palindromic androgen response element (ARE; core sequence 5′-TGTTCT-3′, separated by three nucleotides) located within promoter regions of AR target genes. Next to the DNA binding domain is a small hinge domain, which contains a nuclear localization signal (NLS), and separates the DNA binding domain from the ligand binding domain [27].

In the cytoplasm, AR is bound by a number of chaperone proteins, including heat shock protein 90 (Hsp90) as well as immunophilins. Upon ligand (T or DHT) binding to AR in the cytoplasm, a conformational change exposes the nuclear localization signal, leading to nuclear translocation through the interaction with importin-α protein. Once inside the nucleus, ligand bound AR dimerizes and binds the ARE in promoter regions of AR-target genes, resulting in transcriptional activation or repression, depending on the recruitment of co-activators or repressors, respectively. Examples of co-regulatory proteins are histone lysine demethylases (KDMs), which bind AR and modulate the transcriptional activity of AR-target genes. In sheep placentomes, for example, KDMs have been found to act as binding partners and co-regulators of AR in trophoblast cells [9,22]. This interaction of AR with KDM’s leads to the demethylation of specific lysine residues on histone tails, thereby leading to either transcriptional activation or repression, depending on the specific lysine modification.

To further expand on the breadth and complexity of AR signaling and regulation of genetic pathways, there are at least 20 different AR transcript variants [28]. AR splice variants are derived from post-transcriptional alternative splicing, as well as post-translational modifications to the full-length AR (AR-FL). This can lead to the generation of different-sized, truncated AR proteins with potentially different functions. For example, a prerequisite for AR to regulate target gene transcription is to enter the nucleus. Upon ligand binding, AR-FL exposes an NLS to interactions with importin proteins for translocation through the nuclear pore complex [29]. If an AR isoform does not contain the NLS, nuclear import does not occur, as seen in AR-V1 and AR-V7 (Figure 1), which are primarily present in the cytoplasm [20,30].

**Figure 3:**
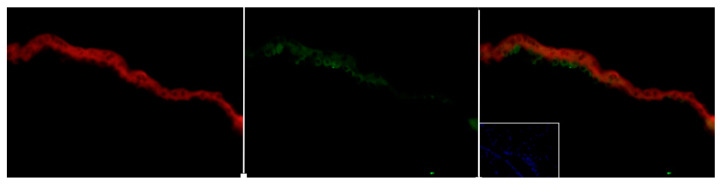
Presence of GPRC6A in human placenta. GPRC6A (green) and hCG (red) staining suggests localization of GPRC6A to syncytiotrophoblast and underlying trophoblast cells. Insert shows a negative control with DAPI.

In addition to signaling through its cytoplasmic/nuclear receptor, it is well-known that steroid hormones can signal through plasma membrane receptors, often referred to as “non-classical” or “non-genomic” signaling [31] In the case of androgens, membrane receptors have been described, including Zrt- and Irt-like protein 9 (ZIP9) [32], transient receptor potential cation channel subfamily M (melastatin) member 8 (TRPM8) and oxoeicosanoid receptor 1 (OXER1) [33], and GPRC6A [34]. GPRC6A is of particular interest as it has been demonstrated to be an important regulator of testicular Leydig cell T synthesis, and also serves as membrane receptor for T. Leydig cells in the testis contain GPRC6A, and studies in mice revealed the existence of a bone–testis axis in which osteocalcin, secreted by osteoblasts, bind GPRC6A on Leydig cells to stimulate steroidogenesis and T synthesis and secretion [35]. GPRC6A signaling following ligand binding can result in the activation of Gαs, Gαi, and Gαq pathways, depending on the tissue [34]. Interestingly, GPRC6A is present in human, mouse and sheep placenta (unpublished data), and localizes to human placental trophoblast cells, including syncytiotrophoblasts (Figure 3). This suggests that androgens can also rapidly activate signaling pathways within trophoblast cells of the placenta. Maternal recognition of pregnancy in humans involves the secretion of human chorionic gonadotropin (hCG) by the syncytiotrophoblasts. Considering that hCG secretion is activated by the cAMP/PKA pathway, and potential coupling of GPRC6A to Gαs, it could be suggested that androgen signaling through GPRC6A is involved with hormone secretion by the syncytial trophoblast layer in the placenta.

## 4. Androgen Signaling in Placenta and during Pregnancy

Since the discovery of steroid receptors in trophoblast cells, the concept of their role in placental development and function has been an object of study. Androgens promote cell growth and differentiation in many tissues, such as prostate cells, through ligand activation of the AR [36]. Studies in mice suggest the role of androgens in the establishment of pregnancy and have a direct impact on implantation. A lack of androgens can delay embryo implantation, whereas excessive levels of androgens led to aberrant gene expression in implantation sites in a mouse model of delayed implantation [37].

Considering the many similar processes that occur between tumorigenesis and placentation (e.g., cell proliferation, invasion, migration), it is not surprising that the process of angiogenesis is similar in the placenta and cancer. Evidence from early studies on first-generation angiogenesis inhibitors such as TNP-470, studied in prostate cancer, implied that impaired angiogenesis is a contributing factor to intrauterine growth restriction of the fetus [38]. Similarly, angiostatin4.5 also reduces murine placental angiogenesis [39]. Androgens have been stablished as fundamental in the processes of endometrial proliferation, decidualization and embryo implantation. Functional studies confirmed that treatment of human primary endometrial stromal cells in vitro with DHT significantly decreased cell proliferation and migration, as well as the AR-dependent inhibition of apoptosis [40]. In vitro studies further reveal that increased levels of androgens affect decidualization, thereby potentially negatively impacting implantation, placentation and establishment of pregnancy [41].

In a sheep model of PCOS, prenatal androgenization leads to gross morphological changes to the placenta, with an apparent overgrowth of the fetal portion of the placenta (cotyledon) [21]. Although the functional significance of these gross morphological changes is unclear, molecular analysis reveal that both AR and VEGFA expression were increased in the placenta. Moreover, additional experiments revealed that AR binds to an ARE in the VEGF promoter in ovine placental tissue. These data suggest that AR signaling may play an important role in regulating placental angiogenesis by regulating the expression of angiogenic factors.

## 5. Conclusions

Steroid hormones are important for the establishment and maintenance of pregnancy, and the contributions of progesterone and estrogens are well-known. It has become increasingly evident that not only are androgens (i.e., testosterone) a necessary precursor for the synthesis of estrogen (i.e., 17b-estradiol), but they play a direct role in placental development and function, as well as pregnancy. Like progesterone and estrogens, androgen levels increase in the maternal serum of humans and animals throughout pregnancy, and the placenta appears to be a key contributor. Importantly, the placenta is a target of androgens, as both nuclear/cytoplasmic androgen receptors and membrane receptors are present in the mammalian placenta.

In this review, we provide an overview of our current understanding of androgens and androgen receptors during normal pregnancy, as well as pregnancy disorders associated with impaired placental development and function. The presence of androgen receptors in all four mammalian placental types is highlighted, as well as its potential role in placental development and function. Future studies focusing on the role of androgen receptors in trophoblast cell differentiation and placental development are needed to understand the functional significance of androgen signaling during normal pregnancy, as well as its contribution to pregnancy disorders.
